# Intergenerational Caregiving Patterns, BMI, and Gender Gaps Among the Sandwich Generation in China

**DOI:** 10.1093/geroni/igaa057.1648

**Published:** 2020-12-16

**Authors:** Jingwen Liu, Feinian Chen

**Affiliations:** 1 University of Maryland, college park, college park, Maryland, United States; 2 University of Maryland, College Park, College Park, Maryland, United States

## Abstract

Existing literatures yield established evidence about the heightened stress brought by multiple roles and potential role overload across work-family context, but little is known about the BMI levels of the “sandwich” caregivers within families and the associated gender inequalities. Indeed, the Chinese pivotal generations are exposed to unshared stress and higher health risks considering that intergenerational support still predominates the caregiving patterns for the oldest old and dependent children under current socioeconomic backgrounds. Using 2011 and 2013 waves of China Health and Retirement Longitudinal Study (CHARLS, N = 12186), we examine associations of BMI and intergenerational caregiving patterns among the “sandwich generation” aged 45 to 69. We find that the sandwich generation with at least one parent alive and one grandchild under 16 have higher BMI (24.2, within obesity range) than their counterparts (23.7, within normal range). A higher proportion of females act as caregivers and especially high-intensity caregivers than males, and they also score one-unit higher in BMI than males (23.4). Fixed effect regression results indicate that simultaneous caregiving to both parents and young grandchildren significantly advances individuals’ BMI levels, while no evidence shows similar negative effect of providing care to one generation. Moreover, high-intensity caregiving (1000 hours and above during the past year) is associated with elevated BMI for females but not for males. The gendered caregiving patterns and health implications inform the physical and psychological vulnerability of the pivotal generation and the necessities of gender-specific intervention in middle and later life.

